# IgA-Based Secretory Response in Tears of COVID-19 Patients: A Potential Biomarker of Pro-Inflammatory State in Course of SARS-CoV-2 Infection

**DOI:** 10.3390/pathogens11101098

**Published:** 2022-09-25

**Authors:** Anna Niedźwiedź, Ewa Pius-Sadowska, Miłosz Kawa, Agnieszka Kuligowska, Miłosz Parczewski, Krzysztof Safranow, Krzysztof Kozłowski, Bogusław Machaliński, Anna Machalińska

**Affiliations:** 1 Department of General Pathology, Pomeranian Medical University in Szczecin, Al. Powstańców Wielkopolskich 72, 70-111 Szczecin, Poland; 2 First Department of Ophthalmology, Pomeranian Medical University, Al. Powstańców Wielkopolskich 72, 70-111 Szczecin, Poland; 3 Department of Infectious, Tropical Diseases and Immune Deficiency, Pomeranian Medical University in Szczecin, Arkońska 4 Street, 71-455 Szczecin, Poland; 4 Department of Biochemistry and Medical Chemistry, Pomeranian Medical University in Szczecin, Al. Powstańców Wielkopolskich 72, 70-111 Szczecin, Poland; 5 Department of Constitutional Law, Faculty of Law and Administration of the Jagiellonian University in Krakow, Bracka 12 Street, 31-005 Kraków, Poland

**Keywords:** COVID-19, secretory response in tears, secretory IgA, Luminex, tear film cytokine levels

## Abstract

Mucosal immunity, including secretory IgA (sIgA), plays an important role in the early defence against SARS-CoV-2 infection. However, a comprehensive evaluation of the local immune response in tears in relation to blood antibody reservoirs has not yet been conducted. A total of 179 symptomatic laboratory-confirmed COVID-19 patients were included in this single-centre study. Conjunctival swabs were analysed by a reverse transcription polymerase chain reaction for the detection of SARS-CoV-2 RNA. In parallel, tear samples collected by Schirmer test strips and plasma samples were analysed by ELISA to detect anti-S1 IgA levels. The concentrations of selected inflammatory cytokines in tears were determined by a magnetic bead assay. Anti-SARS-CoV-2 sIgA was present in the tears of 81 (45.25%) confirmed COVID-19 patients, and the tear IgA levels were correlated with the plasma IgA levels (Rs = +0.29, *p* = 0.0003). SARS-CoV-2 RNA in the conjunctival sac was identified in 18 COVID-19 patients (10%). Positive correlations between the tear IgA level and the concentrations of several cytokines TNF-α (Rs = +0.23, *p* = 0.002), IL-1β (Rs = +0.25, *p* < 0.001), IL-2 (Rs = +0.20, *p* = 0.007), IL-4 (Rs = +0.16, *p* = 0.04), IL-5 (Rs = +0.36, *p* < 0.001), IL-6 (Rs = +0.32, *p* < 0.001), IL-8 (Rs = +0.31, *p* < 0.001), VEGF (Rs = +0.25, *p* < 0.001) and GM-CSF (Rs = +0.27, *p* < 0.001) were also found. Quantitative tear film-based sIgA could potentially serve as a rapid and easily accessible biomarker of external mucosal immunity to SARS-CoV-2. The concentration of sIgA is directly related to individual host immune responses to SARS-CoV-2 infection.

## 1. Introduction

A detailed characterization of the antibody response and an evaluation of its clinical value in COVID-19 patients is important for diagnosis, antiviral treatment, epidemiological investigations and vaccine development. IgA production against the SARS-CoV-2 spike protein early in infected patients might be related to the severity of COVID-19 [1]. Secretory IgA antibodies are present in human mucosal secretions such as tears, milk and saliva to provide protection against infection through IgA-dependent local protective factors [2]. However, relatively little is known about immunity to SARS-CoV-2 at the ocular surface, even though this may be an important entry portal [3,4,5,6]. Thus, there is a need for laboratory serological assays that can measure local antibody responses at the ocular surface for the identification of seroconversion in COVID-19 patients.

Evidence-based epidemiology studies of SARS-CoV-2 infection have shown that the available estimates of infection prevalence largely depend on the availability of molecular and antibody persistence testing and the extent of the tested population. Therefore, the main strategy for controlling the pandemic has depended on testing as many individuals as possible to avoid the risk of transmission to other patients and health care professionals. Unfortunately, not all of the general population has access to rapid testing. The currently available sampling procedures are technically demanding and require the collection of a biological sample from the upper respiratory tract [7]. Moreover, respiratory sample collection can cause discomfort and pain to patients and carries a high risk of virus transmission to health care workers. Recently, Sabage et al. proposed the use of Schirmer strips and conjunctival swabs as methods of tear sample collection for molecular testing [8]. Beltram et al. observed that Schirmer strip wetness has a strong influence on the amount of tear volume absorbed and the volume recovered from these strips [9]. The total protein content in tears collected by Schirmer strips is also influenced by the tear collection method used [9,10]. The Schirmer strip test is routinely used in the ophthalmologic assessment of dry eye syndrome. Therefore, this method may offer the opportunity to routinely evaluate the concentration of SARS-CoV-2-specific immunoglobulins and inflammatory cytokines in tears, principally in the early symptomatic stages of COVID-19.

The main purpose of this study was to analyse conjunctival secretory anti-SARS-CoV-2 IgA in the tears of COVID-19 patients with an emphasis on characterizing the local immune response depending on the severity of SARS-CoV-2 symptoms. To this end, we validated a commonly used serological immunoassay for the quantification of anti-SARS-CoV-2-specific human antibodies in tears and correlated the obtained values with plasma levels in the same cohort of patients. In addition, we characterized the relationship between IgA levels in tear samples and the presence of SARS-CoV-2 RNA in conjunctival swabs, ocular symptoms, COVID-19 severity and the tear fluid concentrations of several cytokines and growth factors. To our knowledge, there are no reports of a comprehensive analysis of anti-SARS-CoV-2 IgA in the conjunctival sac in parallel with an assessment of the systemic IgA response and local inflammation status.

## 2. Materials and Methods

### 2.1. Study Group

This retrospective cohort study included 179 patients diagnosed with COVID-19 in the Department of Infectious, Tropical Diseases and Immune Deficiency of Pomeranian Medical University in Szczecin, Poland between June and December 2020. The group was not subjected to SARS-CoV-2 immunization. All participants underwent a general health examination and completed a detailed ophthalmological health questionnaire. The diagnosis was based on positive reverse transcription polymerase chain reaction (RT–PCR) results for viral nucleic acids of SARS-CoV-2 obtained from nasopharyngeal swabs. The patient was considered positive when at least 2 out of 3 analysed SARS-CoV-2-specific genes (ORF1ab, N, S) had Ct values ≤ 37. Patients with COVID-19 were prospectively enrolled into the ophthalmologic COVID-19 subgroup study cohort. All patients underwent tear sampling and blood collection to determine the concentrations of selected inflammatory cytokines and SARS-CoV-2-specific immunoglobulin A (IgA). Additionally, conjunctival swabs for SARS-CoV-2 detection were collected from all subjects recruited for the study. The collections were performed less than 24 h after admission to the emergency unit. The collected data were used to study the different aspects of ocular immunity for the course of the COVID-19 disease. Thus, the viral RNA and cytokine level data, at least for some of the patients, have been published elsewhere [11,12], using different methods of result comparison analysis.” An informed consent form in accordance with the Declaration of Helsinki was signed by all patients before the study enrolment.

### 2.2. Medical Examination and Detailed Questionnaires

All patients were interviewed to collect information on the presence and duration of typical COVID-19 signs and symptoms such as a fever, dyspnoea, cough, common cold, sore throat, fatigue, chest pain, smell/taste abnormalities, headache, diarrhoea and body aches. The medical examination focused on the respiratory and cardiovascular systems. According to the disease severity classification given in the official guidelines of the Polish Association of Epidemiologists and Infectiologists (PAoEaI) [13,14], patients were divided into four groups (stages 1–4). Stage 1 included patients who were asymptomatic or mildly symptomatic, had an oxygen saturation of at least 95% and did not require hospitalization due to COVID-19. Stage 2 included symptomatic patients with an oxygen saturation lower than 95% who required hospital admission. Stage 3 included patients who started to develop respiratory failure and had an oxygen saturation less than 90%. Stage 4 included critically ill patients who developed acute respiratory distress syndrome (ARDS) and who were in need of mechanical ventilation and ICU treatment. A detailed ophthalmological health questionnaire collected information about the presence and severity of ocular symptoms and their duration at the time of examination in the emergency unit and in the preceding 7 days. Eye-COVID scores (ECSs) were calculated as in previous studies [11,12].

### 2.3. Tear Sample and Conjunctival Swab Collection

The tear samples and conjunctival swab collection and analysis were performed according to a previously developed protocol [11,12] and took place at the time of hospital admission. The concentration of SARS-CoV-2-specific IgA and several inflammatory cytokines (TNF-α, IL-1β, IL-2, IL-4, IL-5, IL-6, IL-8, IL-10, IL-12 p70, GM-CSF, VEGF and IFN-γ) were measured in tear fluid, while the detection of SARS-CoV-2 was performed with conjunctival swabs collected from the lower conjunctival sac. 

### 2.4. Plasma Collection

The peripheral blood samples were collected at the time of hospital admission to assess the plasma level of SARS-CoV-2-specific IgA antibodies. The peripheral blood samples (~5 mL) which were collected in EDTA tubes were centrifuged (2000 rpm, 10 min). The plasma was transferred to a new tube and centrifuged again under the same conditions. The plasma samples were then stored at −80 °C. 

### 2.5. Serological Assay for Specific Anti-SARS-CoV-2 IgA Antibody Detection

**Figure 1:**
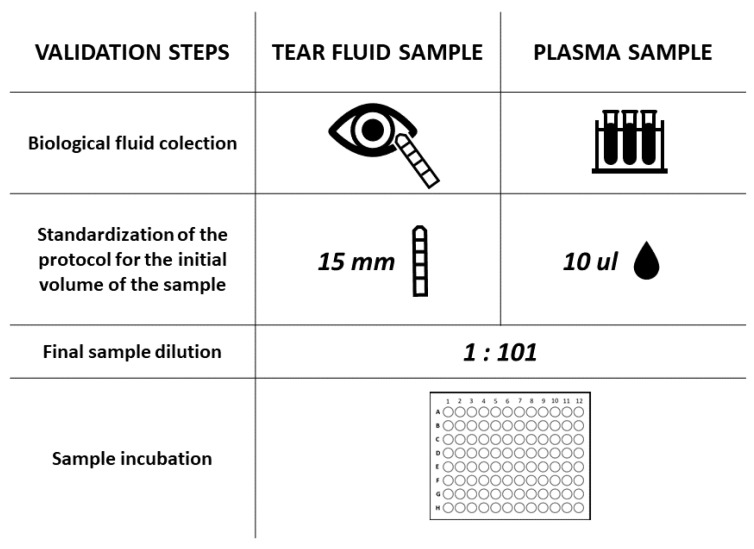
Scheme of the validation of a commonly used serological immunoassay for quantification of IgA anti-SARS-CoV-2 specific human antibodies in tears.

The presence of anti-SARS-CoV-2 IgA in the tears and plasma was evaluated by a CE-IVD ELISA (Euroimmun, Lubeck, Germany) designed to detect IgA against the virus S1 protein. This test was previously reported to have a high specificity and sensitivity for the detection of anti-S1 IgA in serum/plasma samples (greater than 95%) [1,15] and did not demonstrate cross-reaction with other common human coronaviruses. First, the reagent wells on the microplate strips, which were previously coated with recombinant SARS-CoV-2 spike protein domain S1, were filled with the patient’s diluted tear or blood sample and allowed to incubate. If present, the SARS-CoV-2-specific IgA antibodies bound to the coated antigen. To detect the presence of human-specific IgA antibodies, a second incubation was carried out using an enzyme-labelled anti-human IgA that catalysed a colour reaction due to a conjugated enzyme. The results were evaluated semi-quantitatively by calculating the ratio (R) of the extinction of the control or patient sample over the extinction of the calibrator. The calibrator extinction value defines the upper normal range for uninfected individuals (cut-off) recommended by EUROIMMUN. Values higher than the given cut-off are considered positive, and values below the cut-off are negative. The R factor indicates how many times the concentration of antibodies in the test sample is below or above the antibody cut-off value, above which the result is positive. As there is no quantified international reference serum for the assessment of IgA anti-SARS-CoV-2 antibodies, a calibration was performed using Ratio factors, which are a relative measure of serum or plasma antibody concentration. To optimize the manufacturer’s protocol for the in-house evaluation of IgA levels in tears, the tear samples were tested under the same reaction conditions and reagent ratios that were recommended for plasma. In both cases, 10 µL of sample was used as a starting volume. For tears, 10 µL corresponded to 15 mm in height of the Schirmer strip according to a previously described linear relationship that plotted millimetres adsorbed by the Schirmer strip versus the microlitres of collected tears [16]. Both the blood plasma and the tear fluid samples were analysed at a dilution of 1:101. However, in the case of tears, obtaining the correct dilution was a two-step process designed by our team, taking into account the prior elution of the tears from the Schirmer strip in PBS. Following the manufacturer’s instructions, the level of positivity was calculated as the R between the absorbance values of the sample and the calibrator at OD = 450 nm (R < 0.8, negative; 0.8 < R < 1.1, weakly positive; R ≥ 1.1, strongly positive). The validation process is presented in Figure 1.

### 2.6. Luminex Assay 

The concentrations of TNF-α, IL-1β, IL-2, IL-4, IL-5, IL-6, IL-8, IL-10, IL-12 p70, GM-CSF, VEGF and IFN-γ in tear fluid were measured by multiplex fluorescent bead-based immunoassays (Luminex Corporation, Austin, TX, USA) using a commercial R&D Systems Luminex Performance Human High Sensitivity Cytokine Magnetic Panel A (R&D Systems, Minneapolis, MN, USA). One hundred microlitre aliquots of the standard, control and sample were added to the plate together with 25 µL of multiplex antibody capture microparticle solution, and the plate was incubated with agitation for 3 h at room temperature. Subsequently, each well was washed 3 times with 100 µL of wash buffer using a hand-held magnet. Fifty microlitres of detection antibody cocktail was then pipetted into each well, and the plate was sealed and incubated at room temperature for 1 h on a shaker. The wash was then repeated and 50 µL of streptavidin –phycoerythrin mixture was added to each well and incubated with agitation for 30 min in the dark. Finally, after washing, the microspheres in each well were resuspended in 100 µL of wash buffer and shaken at room temperature for 5 min. The plate was then detected on a Luminex 200 analyser, and the analyte concentrations were determined from five different standard curves of the median fluorescence intensity (MFI) vs. protein concentration.

### 2.7. Identification of COVID-19 Positive Patients 

#### 2.7.1. Viral RNA Isolation

The viral RNA isolation was performed using the MagMAX Viral/Pathogen II Nucleic Acid Isolation Kit (Thermo Fisher Scientific, Mississauga, ON, Canada) according to the manufacturer’s protocol. Two hundred μL of each sample was added to the designated sample well and mixed with 5 μL of proteinase K, 5 μL of MS2 phage control, 265 μL of binding buffer and 10 μL of magnetic beads. Two hundred μL of nuclease-free water was also pipetted into the negative control well in the sample plate. Furthermore, 3 processing plates (KingFisher 96 Deep-Well Plate, Thermo Fisher Scientific, Mississauga, ON, Canada) with Wash 1 Solution (500 µL per well), Wash 2 Solution—80% ethanol (1000 µL per well) and Elution Solution (50 µL per well) were also prepared. MagMAX Viral/Pathogen nucleic acid isolation was processed using an automated KingFisher Flex instrument (Thermo Fisher Scientific, Mississauga, ON, Canada).

#### 2.7.2. qRT–PCR Assays for Detecting SARS-CoV-2 RNA 

The RT–qPCR assays for the detection of SARS-CoV-2 RNA were performed using a QuantStudio 5 PCR instrument and a TaqPath COVID 19 CE IVD RT PCR Kit (Thermo Fisher Scientific, Mississauga, ON, Canada) according to the manufacturer’s protocol. The one-step RT–qPCR contained 5 μL of RNA template/ TaqPath COVID 19 Control, 6.25 μL of 4× TaqPath 1 Step Multiplex Master Mix (Thermo Fisher Scientific, Mississauga, ON, Canada), 1.25 µL of COVID-19 Real Time PCR Assay Multiplex and 7.5 µL of nuclease-free water in a total volume of 20 μL. The one-step RT–qPCR program included the RT reaction at 53 °C for 10 min, enzyme activation at 95 °C for 2 min and 40 cycles of PCR amplification at 95 °C for 3 s and 60 °C for 30 s. After RT–PCR was completed, the results were analysed using Applied Biosystems COVID-19 Interpretive Software (Thermo Fisher Scientific, Mississauga, ON, Canada): those tested were considered positive when at least 2 out of 3 analysed SARS-CoV-2 genes (ORF1ab, N, S) had Ct values ≤ 37.

### 2.8. Statistical Analysis 

Because the distributions of the IgA concentrations and other quantitative variables were significantly different from a normal distribution (Shapiro–Wilk test), nonparametric tests were used. The Mann–Whitney test was used to compare variables between the groups, the Wilcoxon signed-rank test was used to compare the plasma and tear IgA levels and the Spearman rank correlation coefficient was calculated as a measure of association between variables. Qualitative variables were compared between the groups with Fisher’s exact test. *p* < 0.05 was considered statistically significant.

## 3. Results

### 3.1. Clinical Characteristics of the Study Group 

A total of 179 SARS-CoV-2-positive symptomatic patients (77 females and 102 males) were included in the study. The mean age of the patients was 57.42 years (SD *=* 14.78). Table 1 presents the clinical characteristics of the study group, including the presence of typical COVID-19 symptoms and the stage of the disease (according to the PAoEaI guidelines) recorded at the time of the examination in the emergency room. 

### 3.2. Detection of Specific Anti-SARS-CoV-2 IgA Antibodies in Tears

**Figure 2:**
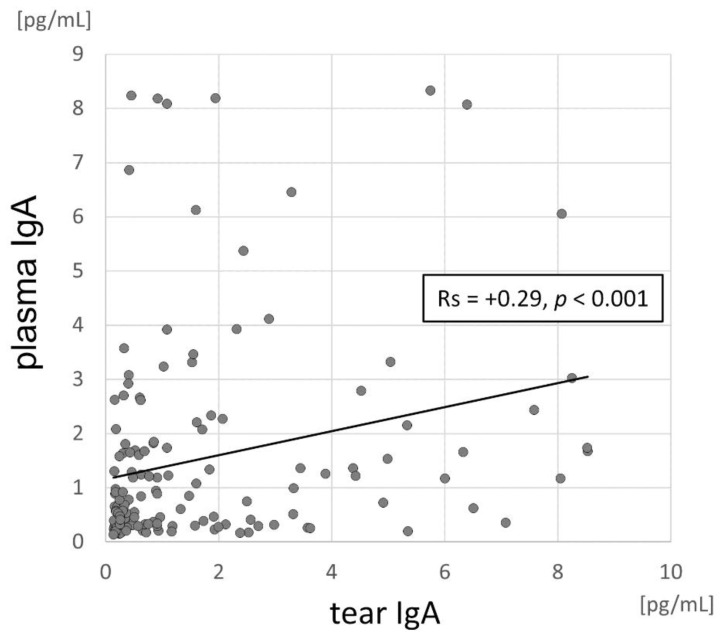
Scatterplot of the correlations between tear fluid and plasma IgA levels of COVID-19 patients.

We semi-quantitatively assessed tear film anti-SARS-CoV-2-specific IgA immunoglobulin using a commercially available assay designed to detect IgA in human plasma or in EDTA-, heparin- or citrate-treated blood plasma samples using the recombinant S1 domain of the SARS-CoV-2 spike protein as antigen. According to the manufacturer’s protocol, 81 (45.25%) of the recruited COVID-19 patients were positive, 15 (8.38%) were borderline and 83 (46.37%) were negative for the presence of IgA in tears. Among tears IgA-positive patients, 53.13% were concurrently plasma IgA-positive, 34.38% were plasma IgA-negative and 12.5% had a borderline score. Regarding tear film IgA-negative patients, 62.34% were also plasma negative, 31.17% were plasma positive and 6.49% had a borderline score. The differences appeared significant (*p* = 0.015). We found no difference between the tear film and plasma-specific IgA levels (median [IQR]: 0.89 [1.72] pg/mL in tear film vs. 0.84 [2.09] pg/mL in plasma; *p* = 0.88). The tear fluid IgA levels were positively correlated with the plasma IgA levels (Figure 2).

### 3.3. Correlation between IgA Levels and Viral Load in the Conjunctival Sac and the Local Inflammatory Response

**Figure 3:**
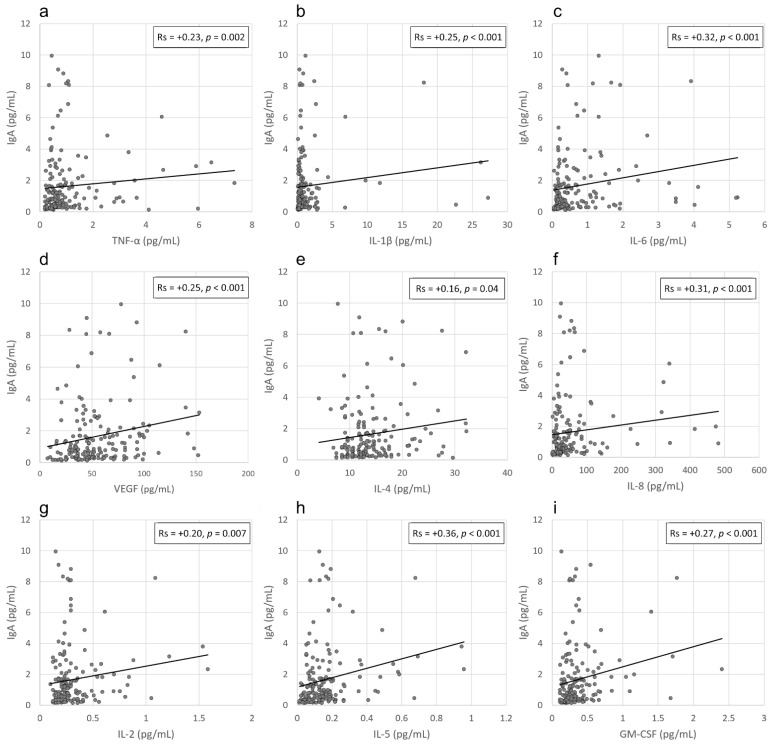
(**a**–**i**). Scatterplots of the correlations between tear fluid IgA levels and the TNF-α (**a**), IL-1β (**b**), IL-6 (**c**), VEGF (**d**), IL-4 (**e**), IL-8 (**f**), IL-2 (**g**), IL-5 (**h**) and GM-CSF (**i**) concentrations in tears of COVID-19 patients.3.4 Correlation between local IgA levels in tears and the severity of eye-related symptoms.

Next, we analysed the local viral activity by detecting SARS-CoV-2 viral RNA in conjunctival secretions and compared it with the local ocular immune response induced by SARS-CoV-2 antigens. This analysis was compared between COVID-19 patients with either a positive or negative conjunctival swab detection. SARS-CoV-2 RNA was identified in conjunctival swabs from 18 patients (10%). We found no difference in IgA levels between the patients positive and negative for SARS-CoV-2 in the conjunctival sac (mean ± SD: 1.82 ± 2.66 pg/mL and 1.68 ± 2.01 pg/mL, respectively; *p* = 0.71). Accordingly, 33.33% of patients positive for IgA in their tears had a positive viral detection on the ocular surface, while 46.25% had negative conjunctival swab results (*p* = 0.32). We then investigated the potential relationship between the local anti-SARS-CoV-2 IgA level and the local immune response. For this purpose, we analysed the levels of selected cytokines in the tears of COVID-19 patients and found positive correlations between tear fluid IgA levels and the TNF-α (Rs = +0.23, *p* = 0.002), IL-1β (Rs = +0.25, *p* < 0.001), IL-2 (Rs = +0.20, *p* = 0.007), IL-4 (Rs = +0.16, *p* = 0.04), IL-5 (Rs = +0.36, *p* < 0.001), IL-6 (Rs = +0.32, *p* < 0.001), IL-8 (Rs = +0.31, *p* < 0.001), VEGF (Rs = +0.25, *p* < 0.001) and GM-CSF (Rs = +0.27, *p* < 0.001) concentrations in tears (Figure 3a–i). In the next step we determined potential correlations between viral load in the conjunctival sac and the tear fluid cytokine levels and found no significant relationships between those parameters.

Next, we assessed whether tear film IgA levels during SARS-CoV-2 infection correlate with ocular symptoms in COVID-19 patients. We analysed the presence and duration of specific eye-related symptoms among the patients with and without the presence of specific anti-SARS-CoV-2 IgA antibodies in their tears (Table 2). We found no relationship between the type of ocular signs and the local anti-SARS-CoV-2 IgA antibody level. Similarly, the duration of ophthalmologic symptoms was not correlated with the secretory IgA antibody levels in the collected tear samples with one exception: misty vision during the preceding 7 days was found to correlate with IgA positivity (*p* = 0.03). Regarding correlations with the gender of the patients, we found no association between sex and IgA tear or plasma levels (*p* > 0.2), but there was a significant association between the gender and symptoms; there was a higher prevalence of eye burning during the preceding 7 days in females than in males (11% vs 2%, *p* = 0.020).

### 3.4. Correlation between Local IgA Levels and COVID-19 Severity

**Figure 4:**
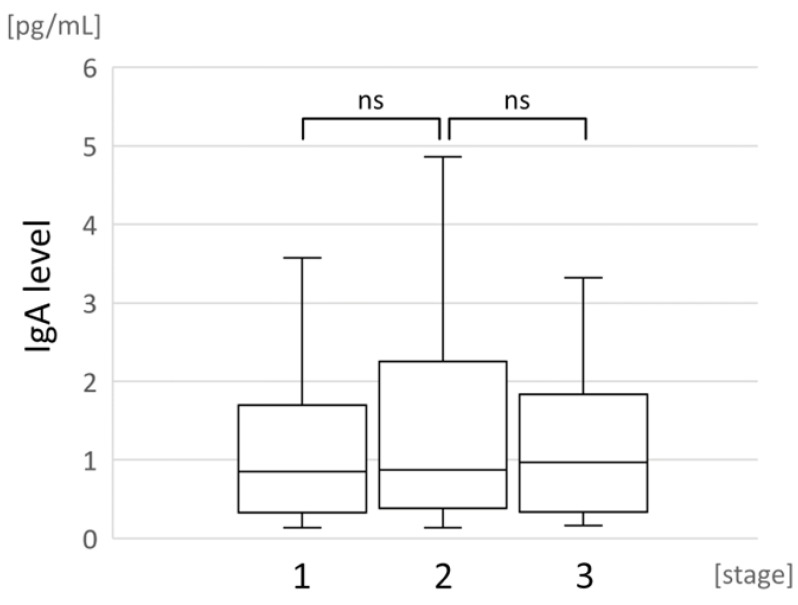
Boxplots showing tear IgA levels in different disease stages recorded at the time of the examination during emergency room admission. ns—non statistic.

No correlation was observed between typical COVID-19 signs and symptoms and the IgA antibody levels. The IgA antibody levels in tear fluid samples were not significantly affected by the disease stage (Figure 4) or the application of specialized treatments such as oxygen therapy or ICU treatment in response to multiple organ failure. The tear film IgA positive and negative groups did not differ in age (median = 58 years vs. median = 60 years, respectively; *p* = 0.22) and sex (*p* = 0.21).

## 4. Discussion

Although the humoral immune response is important for viral containment, the local antiviral response in tear fluid is still poorly understood. IgA antibody constitutes 15–20% of the total immunoglobulins circulating in human serum. IgA is present in blood and in external fluid secretions of the mucosa and conjunctiva. The essential biological function of IgA is to protect the body against molecular antigens that can be absorbed through the mucosal surface. This immunoglobulin constitutes the first line of defence against infection by blocking viral adhesion to epithelial cell receptors [17]. Moreover, human serum immunoglobulin A has strong immunomodulatory properties because it modulates the release of cytokines and chemokines from immune-related cells [18]. In the first stage of COVID-19, several body sites have a potent antigen-neutralizing IgA response, including the saliva, tears and bronchoalveolar fluid [19,20]. Thus, the SARS-CoV-2 neutralization that occurs in the first weeks following COVID-19 symptoms onset is more closely associated with secretory IgA levels than IgM or IgG levels [21].

Thus, the main objective of the present study was to evaluate the concentration of SARS-CoV-2-specific immunoglobulin A and selected inflammatory cytokines and chemotactic growth factors in tears from symptomatic COVID-19 patients using classic Schirmer strips. These strips are commonly used in the ophthalmologic examination of tear volume and composition. To validate the credibility of using commercially available blood-based serological assays for tear film analysis as a COVID-19 diagnostic, we studied the relationship between the levels of anti-SARS-CoV-2 IgA antibodies in tears and in blood in the early phase of infection. In addition, we investigated whether there was a relationship between anti-SARS-CoV-2 IgA antibody presence and the severity and duration of ocular signs and symptoms of COVID-19 patients.

The results showed that anti-SARS-CoV-2 IgA antibody titres were similarly distributed in tears and blood plasma in the early phase of COVID-19. Importantly, the anti-SARS-CoV-2 IgA level in tear film was positively associated with the blood serum IgA concentration. This indirectly confirmed the potential usability of commercially available serological immunoassays for the detection and evaluation of immunoglobulin concentrations in tear film. Previous studies have described the presence of a local IgA-based immune response against SARS-CoV-2 in tears and conjunctival fluid [22,23,24]. However, the current study is the first demonstration of the direct positive correlation between IgA levels in the tear fluid and blood. Our data confirm the importance of the eye as a site of SARS-CoV-2 replication and indicate the detection of antibody levels in tears as a non-invasive surrogate for blood plasma in monitoring host immune responses in an in vitro laboratory setting.

In contrast to the findings of Mahmoud et al., we did not find a significant correlation between anti-SARS-CoV-2 IgA antibodies and the severity and duration of ocular symptoms in COVID-19 patients [24]. The stage and severity of COVID-19 are important factors associated with the expression of secretory IgA, but variations in the immune system and genetic differences among patients may have led to differences in the humoral response to SARS-CoV-2 detected by different research groups. We also found no relationship between the presence of SARS-CoV-2 viral RNA in the conjunctival sac and the local IgA response induced by SARS-CoV-2 antigens. In our study, SARS-CoV-2 RNA was identified from conjunctival swabs in only 10% of the confirmed COVID-19 patients. This is in agreement with a previous study that found that the virus was detectable at the ocular surface in the conjunctiva in a small number of COVID-19 patients [25]. Indeed, secretory IgA has been shown to be a significant component in mediating mucosal immunity as a first-line defence against SARS-CoV-2 infection. Mucosal immunity represents an important protection against pathogenic microorganisms and constitutes an immune barrier that neutralizes viruses before they reach and bind to epithelial cells [17].

Recent studies have also found that secretory IgA is associated with local immune response activation. IgA complexes with CD71 to initiate an inflammatory feedback loop by inducing its enhanced expression. IgA also induces cell proliferation, cytokine/chemokine production (including primary proinflammatory cytokines such as IL-1, IL-6 and TNF-α) and chemotactic activity towards leukocytes [26]. In humans, IgA reportedly has both proinflammatory and anti-inflammatory roles [18]. Olas et al. reported that plasma-derived IgA downmodulates the release of the proinflammatory cytokines IL-6 and tumour necrosis factor (TNF)-alpha from LPS-stimulated monocytes and upregulates the release of IL-1 receptor antagonist (IL-1RA) from resting monocytes [18]. Immunoglobulin A also plays a critical role in immune homeostasis by modulating the functions of specialized immune cells. IgA has a immunoregulatory function towards human Th17-type cells, which are the key mediators of a variety of inflammatory and allergic diseases. Saha et al. showed that IgA inhibits the differentiation and amplification of human Th17-type cells and the production of their effector cytokine IL-17A. IgA was also able to suppress IFN-γ responses in human Th17-type cells [27]. The evaluation of cytokines and other inflammation-related parameters is essential for detecting the acute phase of inflammation [28]. Thus, we previously investigated the concentration of inflammatory cytokines, including several interleukins and TNF-α, and other chemotactic growth factors, such as GM-CSF, that are crucial for white blood cell trafficking. We identified a unique profile of several biologically active substances in the tear film of patients with early SARS-CoV-2 infection [11]. In the current study, TNF-α, IL-1β, IL-2, IL-4, IL-5, IL-6, IL-8, VEGF and GM-CSF, were significantly related to anti-SARS-CoV-2 IgA expression in the ocular environment. This relationship indicates that the activation of the local inflammatory response in the conjunctival sac is strongly related to the IgA effector functions in resident immune cells due to the binding and signalling properties of IgA [29]. 

Our work provides a basis for the potential development of a rapid tear collection method for immunologic studies of SARS-CoV-2 infection. This study also demonstrates the use of commercially available serological assays for the unstandardized purpose of evaluating immunoglobulin concentrations in human tear film in a laboratory setting. We demonstrated that our in-house protocol for specific IgA quantification is reliable and efficient for tear-based immunological evaluation in COVID-19 patients. Although a comprehensive approach to evaluate the details of the analytical procedure is desirable, this study lays the foundation for the development of an effective and innovative diagnostic method that would increase the availability of diagnostic tools to address the clinical and epidemiological needs of medical care systems worldwide. 

In summary, our observations suggest potential consequences for the current understanding of SARS-CoV-2 seroepidemiology as well as for basic and applied COVID-19 science. Although they are still at their early stages, our studies may provide further, more specific insights on the exact role of the soluble IgA in COVID-19 disease. Especially, due to the non-invasive nature of accessing conjunctival secretions in humans, we hope that the availability of conjunctiva-specific samples will expand the knowledge on antiviral protection against SARS-CoV-2. In this notion, our preliminary analyses may help to create the new epidemiological modelling strategies for patients care and to provide the new public health policy.

## Figures and Tables

**Table 1:** Clinical characteristics of the study group, including the presence of typical COVID-19 symptoms, recorded at the time of the examination during emergency room admission.

Parameter	Number of Patients	%
Sex (male/female)	102/77	56.98/43.02
*Stage of the disease (according to the PAoEaI guidelines):*		
1	45	25.14
2	98	54.75
3	36	20.11
4	0	0
*Medical history:*		
Need for the hospitalization	134	74.86
*COVID-19 symptoms:*		
Fever above 38 °C	125	69.83
Dyspnoea	82	45.81
Cough	135	75.42
Chest pain	50	27.93
Smell/taste disorders	64	35.75
Headache	69	38.55
Diarrhoea	48	26.82
Pneumonia	161	90.96
Conjunctivitis *	2	1.12

* redness + at least two other ophthalmic symptoms, i.e., swelling of the conjunctiva, itching/burning, sand sensation under the eyelid or watering of the eyes.

**Table 2:** Ophthalmological characteristics of patients with and without the presence of specific anti-SARS-CoV-2 IgA antibodies in tears at the time of admission into the emergency room. Statistically significant *p* values are shown in bold.

	Ophthalmic Symptom	IgA Positive	IgA Negative	*p*
*% of patients with a given ophthalmic symptom* *at the time of enrolment*	Eyelids swelling	1.30	1.28	1.00
	Eye itching	2.60	3.85	1.00
	Eye burning	3.90	7.69	0.49
	Eye tearing	3.90	10.26	0.21
	Eye redness	3.90	2.56	0.68
	Sand sensation under the eyelid	1.30	3.85	0.62
	Presence of the discharge	2.60	1.28	0.62
	Gluing of the eyelids	3.90	0.00	0.12
	Light sensitivity	2.60	3.85	1.00
	Eye stiffness	0.00	1.28	1.00
	Eye pain	2.60	6.41	0.44
	Visual impairment	2.60	2.56	0.28
	Misty vision	2.60	2.56	1.00
	Blurry vision	3.90	3.85	1.00
*% of patients with a given ophthalmic symptom* *during the preceding 7 days*	Eyelids swelling	2.60	0.00	0.25
	Eye itching	2.60	3.85	1.00
	Eye burning	5.19	6.41	1.00
	Eye tearing	7.79	10.26	0.78
	Eye redness	2.60	5.13	0.68
	Sandy sensation under the eyelid	2.60	2.56	1.00
	Presence of discharge	3.90	1.28	0.37
	Gluing of the eyelids	2.60	2.56	1.00
	Light sensitivity	5.19	2.56	0.44
	Eye stiffness	1.30	1.28	1.00
	Eye pain	2.60	7.69	0.28
	Visual impairment	5.19	2.56	0.44
	Misty vision	6.49	0.00	**0.03**
	Blurry vision	5.19	0.00	0.06

## Data Availability

The data that were used to support the findings of this study are available from the corresponding author upon request.
